# A case report of metastasis of malignant mesothelioma to the retromolar trigone

**DOI:** 10.1186/s12957-016-0942-1

**Published:** 2016-07-20

**Authors:** A. Arslan, C. Ozcakir-Tomruk, E. Deniz, O. Akin

**Affiliations:** Faculty of Dentistry, Department of Oral and Maxillofacial Surgery, Yeditepe University, Bagdat Cad, No: 238, Goztepe, 34728 Istanbul, Turkey; Patomed Pathology Laboratory, Gursel Mah, Darulaceze Cad, Eksioglu Is Merkezi, Kat:3, No: 36, D:4, Okmeydani, Istanbul, Turkey

**Keywords:** Mesothelioma, Metastasis, Oral mucosa, Retromolar trigone

## Abstract

**Background:**

Malignant mesothelioma is a rare and aggressive tumor with a poor prognosis. Distant metastases are very rare, and the oral cavity metastases are exceedingly rare. Only a few cases with metastasis to oral gingiva are reported. To our knowledge, this is the first case report of pleural mesothelioma metastasized to the retromolar trigone.

**Case presentation:**

A 59-year-old male was referred with a painless growth at retromolar trigone area. It had been present for 2 months and had increased in size during this period of time. Past medical history revealed a malignant mesothelioma. Intraoral examination showed a soft, haemorrhagic, ulcerated lesion at the right retromolar trigone area. There was no destruction of the bony architecture. An incisional biopsy was performed under local anaesthesia. Based on the histopathological and immunohistochemical findings, a final diagnosis of metastatic mesothelioma was made. The patient was informed about the possibility of multiple metastases within the body, but he succumbed after 45 days following deterioration of his medical condition.

**Conclusions:**

Biopsy, history of the patient and clinical picture were provided to the clinicians to make an efficient differential diagnosis. Differential diagnosis must be performed with other oral cancers, because the management is totally different.

## Background

Malignant mesothelioma is a malignant neoplasm of mesodermal origin and arises from multipotential mesothelial or subserosal cells of the pleura, pericardium and peritoneum. There are different forms: epithelioid (60 %), sarcomatoid (10–20 %) and biphasic patterns (20–30 %) [1]. Distant metastases are very rare, and the oral cavity metastases are exceedingly rare [2]. An English literature review revealed seven cases on the tongue, two cases of the mandibular gingiva, two cases of the buccal mucosa, one case of the bone associated with the mandibular third molar and one case of the floor of the mouth [3]. Ohnishi et al. [4] reported another four cases of oral gingiva metastasis from diffuse malignant pleural mesothelioma. This is the first case of a metastasis of mesothelioma encountered in the retromolar trigone. Metastases are more common in the jaw bones than the soft tissue. The most common sites for men are the lungs (27 %), kidneys (13 %) and skin (13 %)—for women, the breast (24 %) and genital organs (17 %), followed by the bone (10 %) and kidney (10 %) [5]. Hirshberg et al [6] reported 58 cases (50 males, 7 females) of metastasis to the jaw bone whereas 54 cases (42 males, 7 females) of metastasis to the oral mucosa out of 112 patients having lung cancer. He also reported 36 cases (21 males, 15 females) of metastasis to the jaw bone and 29 cases (19 males, 9 females) of metastasis to the oral mucosa out of 65 patients having kidney cancer [6]. Patients with breast cancer (3 males, 108 females) metastasized in the jaw bone in 91 patients (1 male, 90 females) and to the oral mucosa in 20 patients (2 males, 18 females) [6]. The reason for this gender-dependent metastatic pattern has not completely been elucidated yet. In 90 % of all pleural mesothelioma, asbestos association due to occupational exposure is reported. Most people are in their fourth to seventh decade at the time of diagnosis [5]. The latency between exposure and manifestation takes approximately 20–40 years. Occurrence of the malignant disease typically carries an average survival rate of 9–12 months. In the present case report, a patient with a history of malignant pleural mesothelioma with metastatic disease to the retromolar trigone is presented.

## Case presentation

**Fig. 1:**
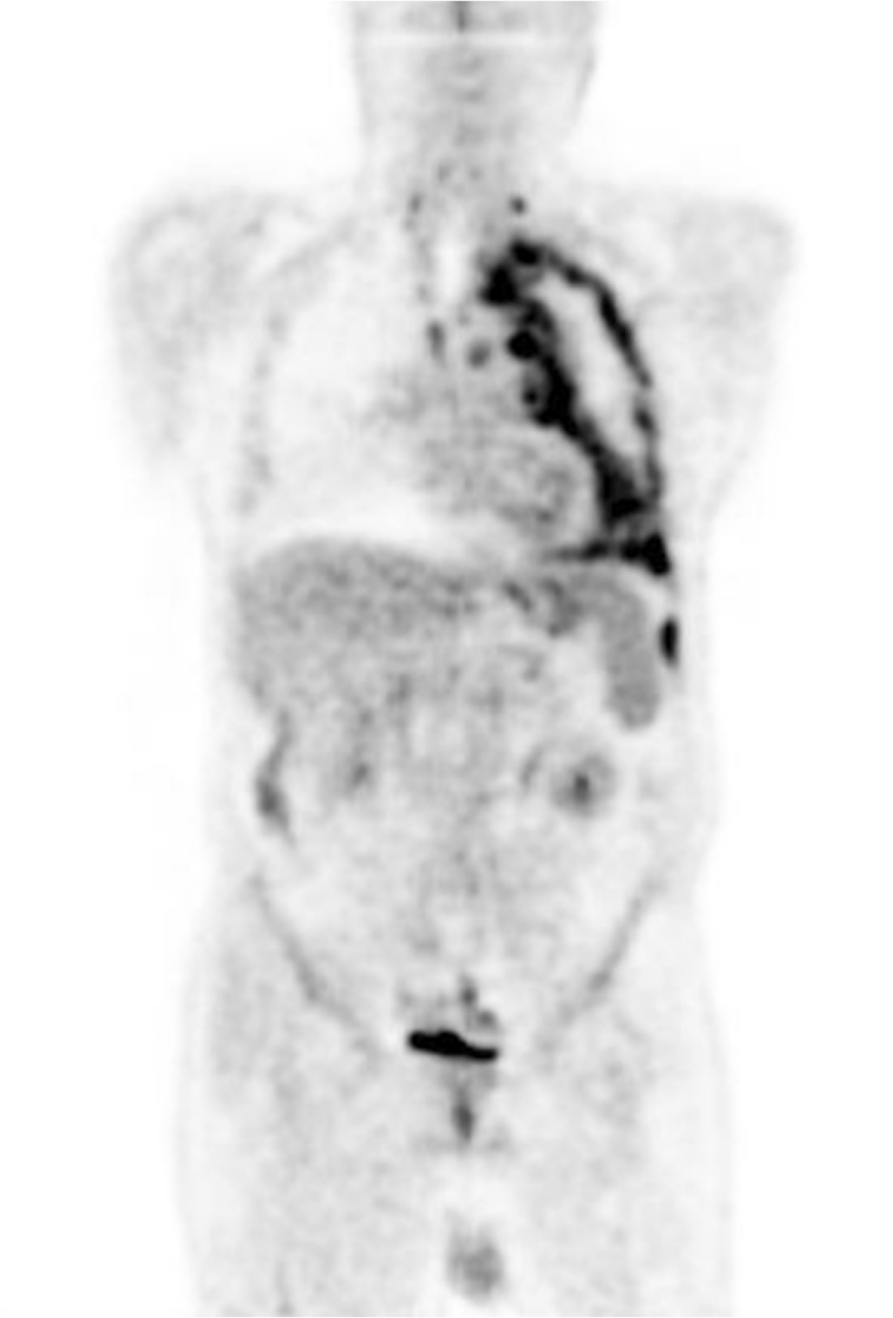
Coronal PET (positron emission tomography) image showed higher FDG (fluorodeoxyglucose) uptake in the left lung and the lymph nodes on the left side

**Fig. 2:**
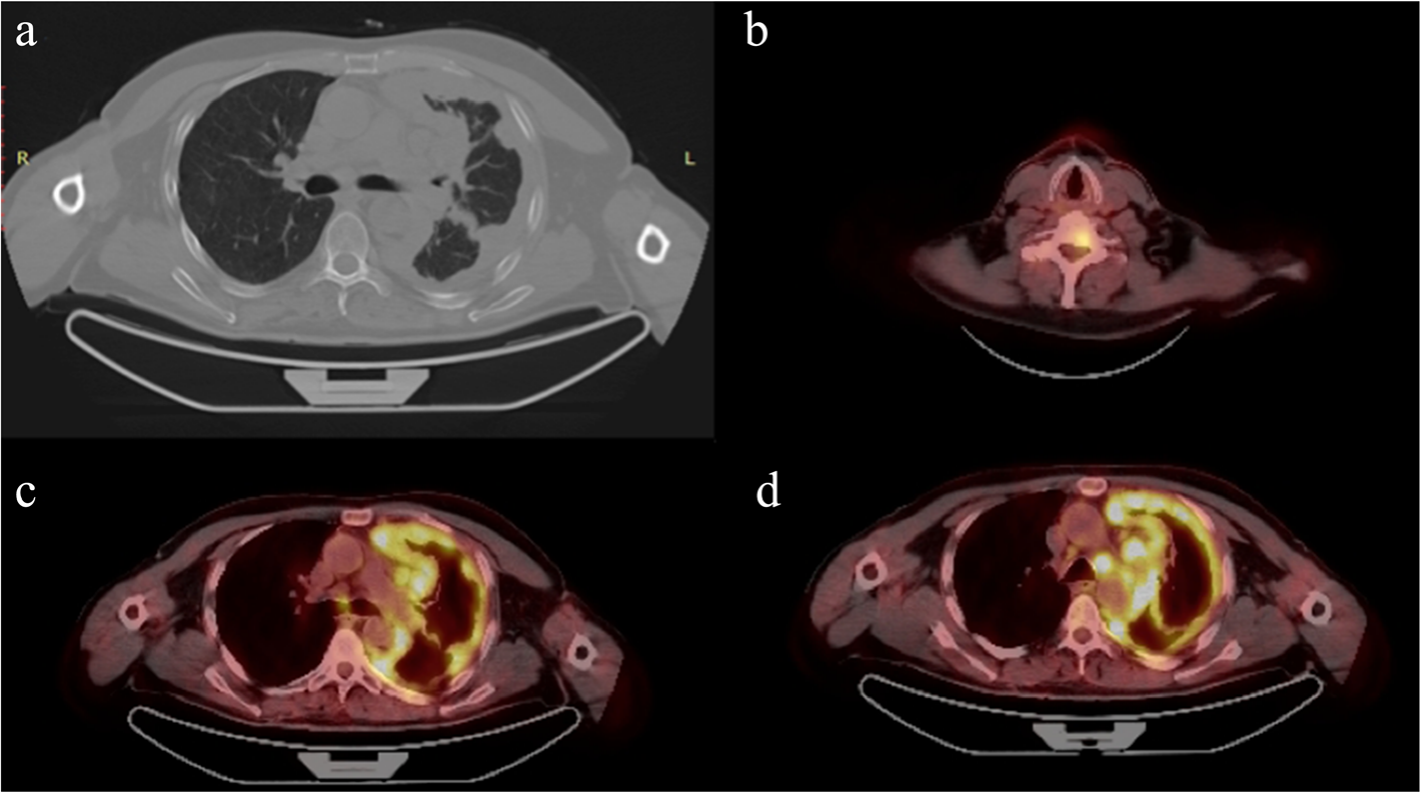
**a** Chest CT presenting mesothelioma. **b**, **c** Axial PET images with higher tracer uptake. **d** Axial PET image indicating C7 vertebrae having a possible metastatic view

A 59-year-old male was referred to the Department of Oral and Maxillofacial Surgery with a painless growth at retromolar trigone area. It had been present for 2 months and had increased in size during this period of time. Past medical history revealed a malignant mesothelioma which was diagnosed in September 2011 and treated with chemotherapy and radiotherapy until February 2014. The patient received 6 cycles of cisplatin (50 mg/100 ml, every 28 days) and premetrexed (500 mg, every 3 weeks) at the beginning. Since the lesion was inoperable, radiotherapy (50 Gy, in 25 fractions) was given after chemotherapy sessions. After radiotherapy sessions, starting from April 2012, the patient received 5 cycles of premetrexed (500 mg, every 3 weeks). A different regimen was given starting from January 2013. Cisplatin (50 mg/100 ml, every 28 days) and gemcitabine (1000 mg/m^2^) were prescribed. Gemcitabine was given on the 1st, 8th and 15th days. He was 71 kg when gemcitabine was started. After 4 months, the patient was 66 kg. Pemetrexed (500 mg, every 3 weeks) was prescribed instead of gemcitabine. The patient received 750 mg pemetrexed every 3 weeks until December 2013. In August 2013, positron emission tomography (PET CT) of the patient was evaluated. Figures 1 and 2 show the location of the tumor on the left lung and also possible metastasis on C7 vertebrae. He was 63 kg on that date. In January 2014, the patient was referred to our department because of the complaint in the oral region.

**Fig. 3:**
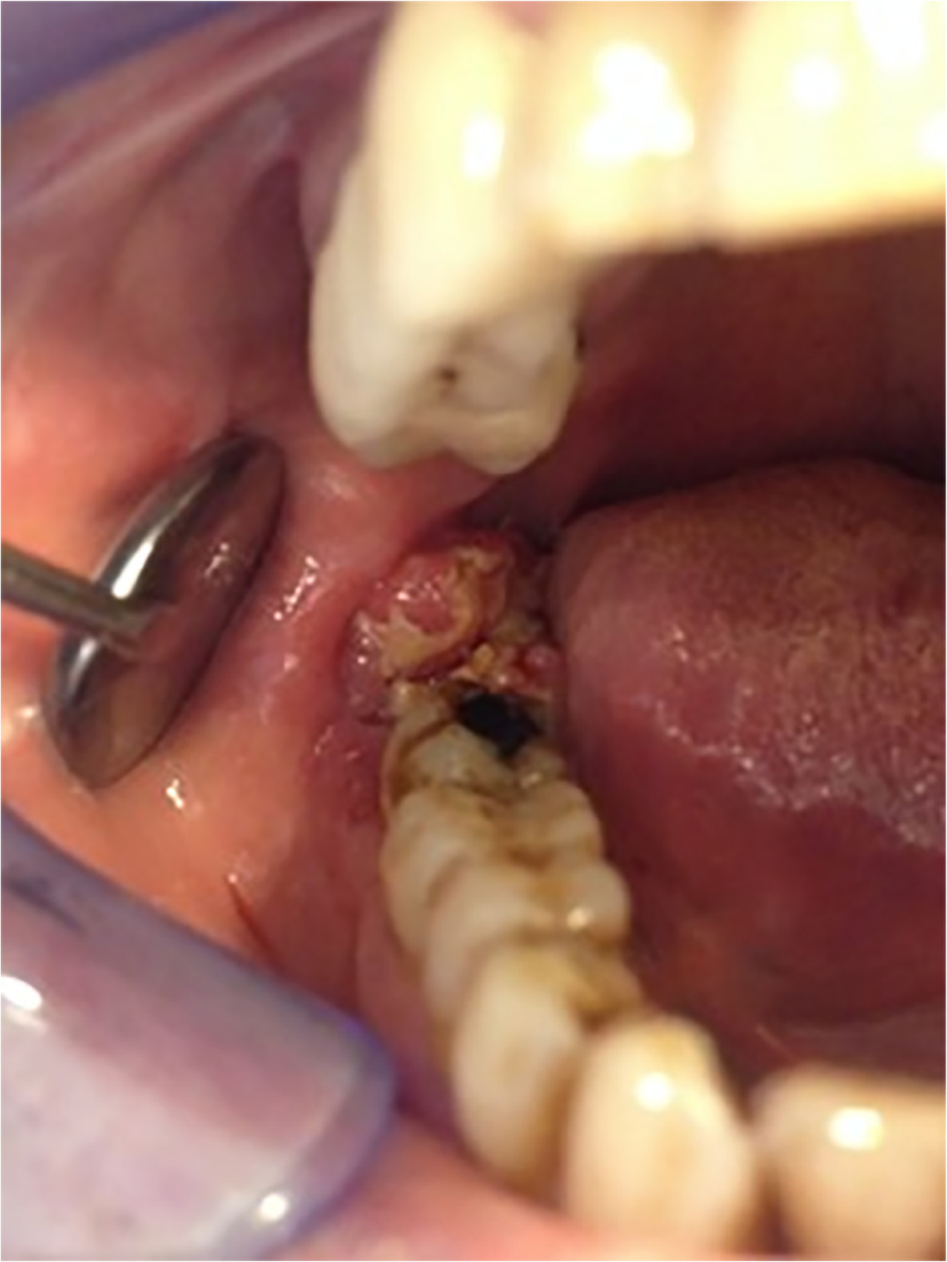
Intraoral view of the patient

**Fig. 4:**
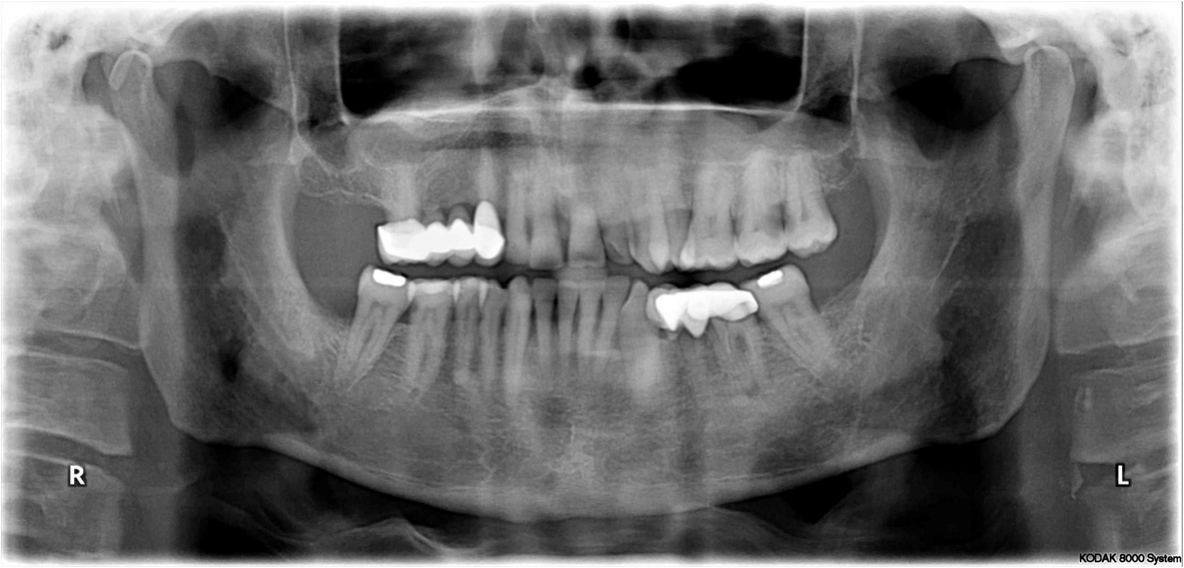
Panoramic radiograph of the patient

**Fig. 5:**
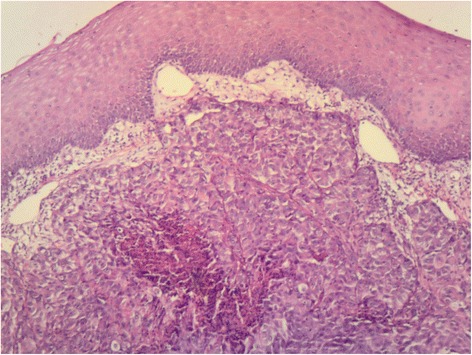
Invasive malignancy at subepithelial area (HE ×40)

**Fig. 6:**
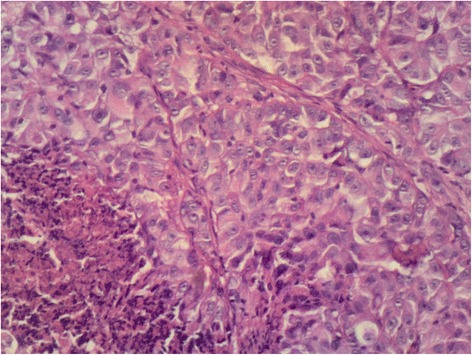
Huge cells with pleomorphic nucleus and narrow cytoplasms

**Fig. 7:**
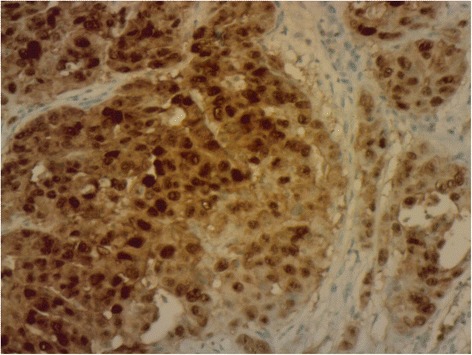
Strong positivity for calretinin on immunohistochemical staining

Intraoral examination revealed a soft, haemorrhagic, ulcerated lesion at the right retromolar trigone area (Fig. 3). Extraorally neither swelling nor lympadenopathy was observed. The innervation of N. trigeminus was balanced on both sides. There was no paresthesia on the lingual nerve. Radiologic examination revealed no destruction of the bony architecture (Fig. 4). An incisional biopsy was performed under local anaesthesia. Haematoxylin and eosin staining showed invasive malignancy at subepithelial area (Fig. 5). Syncytial aggregations composed of huge cells with pleomorphic nucleus, distinctive nucleolus and narrow cytoplasms were also detected (Fig. 6). For immunohistochemical staining, the slides were stained with antibodies to calretinin (Ready to use, NeoMarkers, USA), secondary antibodies and DAB chromogen using standard procedures. Cytoplasmic/nuclear calretinin expression was considered positive. Intensity of staining was graded 3. The tumor cells were strongly positive for calretinin on immunohistochemical staining (Fig. 7). Based on these histopathological and immunohistochemical findings, a final diagnosis of metastatic mesothelioma was made. The patient was informed about the possibility of multiple metastases within the body. He succumbed after 45 days following the deterioration of his medical condition.

### Discussion

Malignant mesothelioma is a rare and aggressive tumor with a poor prognosis. The incidence of this disease is one or two cases per million per year [7]. The diagnosis of the primary malignant mesothelioma is established by clinical, histological, immunohistochemical, and radiological findings. In the present report, calretinin, a mesothelial marker turned out to be strongly positive in tumour cells on immunohistochemical staining, substantiating diagnosis of an epithelioid mesothelioma metastasis. Calretinin, a helix–loop–helix structural domain found in calcium-binding proteins and widely expressed in neural tissue, is expressed in most cases of epithelioid mesothelioma [8]. For radiological investigations, ESMO (European Society For Medical Oncology) recommends using CT scanning of the thorax for the localization of tumor sites and distant metastases or detecting early response to treatment. They recommend also MRI in special situations where tumor delineation is necessary [9]. But the diagnosis of the secondary malignant mesothelioma in the oral cavity, as in the present case, is based on the presence of known history of the tumor. Even with the presence of the primary tumor, it is necessary to make a definite diagnosis of an assumed oral metastases to allow for an appropriate management. Although the clinical behaviour and the view of the primary oral squamous cell carcinoma (OSCC) is unlikely to be misdiagnosed for a metastatic lesion by an experienced surgeon, OSCC is the most likely diagnosis of a persistent mass, without obvious traumatic or infectious origin. Wide surgical excision is often an acceptable treatment for the management of OSCC, whereas in a metastatic mesothelioma case, it will be inappropriate [10]. The current effective standard treatment for mesothelioma is the addition of bevacizumab to pemetrexed plus cisplatin which significantly improves the overall survival [11]. Unfortunately, there was no effective standard treatment for mesothelioma during the follow-up period of the present patient. Therefore, another different regimen of chemotherapy was prescribed. Although the cisplatin plus pemetrexed therapy of the present patient was the standard of care in the first-line treatment, the prognosis was poor and the patient succumbed to the disease.

Malignant mesotheliomas frequently recurred locally in the chest and abdomen. Metastases occur at a relatively late stage of the disease. Kannerstein and Churg [12] reported metastases in 18/50 autopsy cases. Distant metastases are very rare, especially in the oral region. Primary breast carcinomas are the most common types of tumour to metastasize to the jaw bones while primary lung carcinomas are the most common types of tumour to metastasize to the oral soft tissue [13]. These oral metastases represent about 1 % of oral malignances, and there are more cases reported arising in the jaw bones than in the oral soft tissues with a ratio of approximately 2:1 [14]. The case presented in this report is only the first report of a mesothelioma metastatic to the retromolar trigone. Although Kruse et al. [13] and Hirshberg et al. [6] reported that metastases of mesothelioma, although rare, tend to involve the oral soft tissues, especially the tongue. Sinon et al.[3] reported that metastases to oral region are mostly seen in the tongue and the attached gingiva. One possible explanation for the tendency towards the tongue and the attached gingiva might be the rich capillary network, especially where chronically inflamed gingiva trap malignant cells and the fragmented basement membranes of proliferation capillaries allow easier penetration by malignant cells than in more mature blood cells [15].

## Conclusions

This case report points out the relevance of biopsy to all new growing lesions, even in uncommon anatomical sites, whenever a history of mesothelioma is on record. Biopsy, history of the patient and clinical picture were provided to the clinicians to make an efficient differential diagnosis. An accurate diagnosis is very important to avoid inappropriate futile surgery and help maintain the patients’ quality of life with the aim of improving the oral condition.

## Abbreviations

FDG, fluorodeoxyglucose; N. trigeminus, nervus trigeminus; OSCC, oral squamous cell carcinoma; PET, positron emission tomography
